# Occult ovarian carcinoid in pregnancy: a case report and literature review

**DOI:** 10.3389/fonc.2026.1793002

**Published:** 2026-06-10

**Authors:** Yong Zhu, Yanqing Wu, Ying Chen

**Affiliations:** 1 Department of Obstetrics and Gynecology, People’s Liberation Army General Hospital of Central Theater Command, Wuhan, Hubei, China; 2 The 970th Hospital of the Joint Logistics Support Force of the Chinese People’s Liberation Army, Weihai, Shandong, China

**Keywords:** adnexectomy, occult tumor, ovarian carcinoid, pregnancy, trabecular carcinoid

## Abstract

Carcinoid tumor is a rare neuroendocrine neoplasm, and primary ovarian carcinoid is extremely uncommon. Ovarian carcinoid during pregnancy is exceptionally rare and diagnostically challenging. We report the case of a 30-year-old primigravida who underwent cesarean section for fetal distress, during which a left ovarian cyst was incidentally found. Pathological examination revealed a mature cystic teratoma with carcinoid (trabecular type). The patient underwent laparoscopic adnexectomy six weeks after cesarean delivery with curative intent. Final pathological staging was FIGO IA. Maternal and neonatal outcomes were favorable, with no evidence of recurrence during 18 months of follow-up. This case highlights the clinicopathological features, diagnostic challenges, management strategies, and prognosis of ovarian carcinoid during pregnancy and aims to enhance clinical awareness.

## Introduction

Ovarian carcinoid is a rare germ cell tumor that is often associated with mature cystic teratoma (1). The incidence of ovarian tumors in pregnancy ranges from 0.15% to 5.7%, with malignancies accounting for 1%–3% of cases (2). Ovarian carcinoid during pregnancy is frequently asymptomatic, lacks specific biomarkers or typical imaging findings, and is often discovered incidentally during surgery (3). Herein, we report a case of occult ovarian carcinoid detected during cesarean section and review the relevant literature to discuss clinical management.

## Case report

A 30-year-old primigravida at 39^+6^ weeks of gestation was admitted for cesarean section due to fetal tachycardia, oligohydramnios, and elevated D-dimer levels suggestive of fetal distress. During surgery, a left ovarian cyst was incidentally identified.

### Patient history

The patient had regular menstrual cycles. Eight prenatal ultrasounds revealed no adnexal abnormalities. Non-invasive prenatal testing indicated a duplication on chromosome 1q, and amniotic fluid chromosomal microarray analysis revealed a variant of uncertain significance. Gestational diabetes mellitus (A1 type) was diagnosed using an oral glucose tolerance test. Family history was negative for any malignancy, including breast, ovarian, colorectal, and neuroendocrine tumors.

### Prenatal ultrasound findings

Despite the presence of a 6 cm ovarian teratoma that was subsequently identified during cesarean section, all eight prenatal ultrasounds failed to detect any adnexal mass. This can be explained by several factors. First, during late pregnancy, the enlarged uterus displaces the adnexa upward and posterolaterally, making ultrasound visualization technically difficult. Second, the teratoma itself had no solid component on ultrasound, appearing as a complex cyst with fat and calcification, and conventional ultrasound has low sensitivity for detecting solid foci smaller than 1 cm. Third, the carcinoid focus, measuring only 1.2 mm, was entirely embedded within the cyst wall and therefore did not result in a detectable solid mass. Consequently, the tumor remained completely occult during the prenatal period.

### Diagnosis

**Figure 1:**
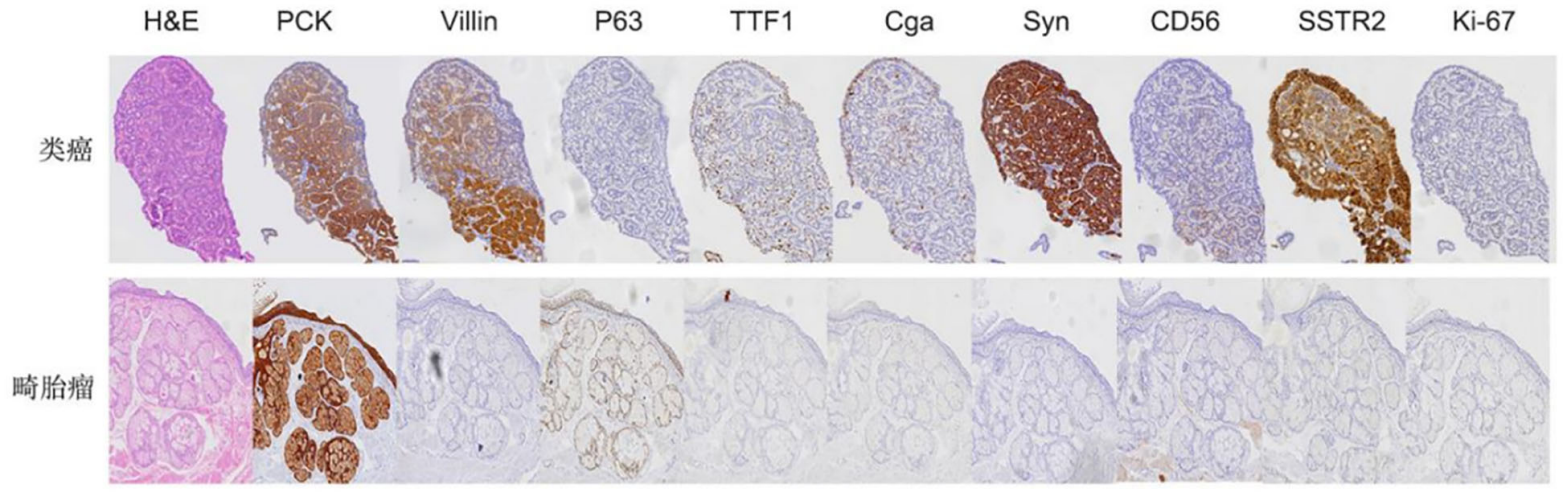
Microscopic findings on H&E staining and immunohistochemical staining in a mature cystic teratoma with a carcinoid tumor (trabecular type).

The preoperative diagnosis included term pregnancy, suspected fetal distress, and gestational diabetes mellitus A1. Intraoperatively, a left ovarian cyst measuring approximately 6.0 × 4.0 × 3.0 cm) was identified. Postoperative pathological examination demonstrated a mature cystic teratoma with carcinoid (trabecular type). Immunohistochemical staining showed Syn(+), CD56(+), SSTR2(+), CgA(focal+), and Ki-67 ≈1% (**Figure 1**). The carcinoid focus measured approximately 1.2 mm in maximum diameter, explaining its “occult” nature and undetectability on prenatal ultrasound.

### Treatment

**Figure 2:**
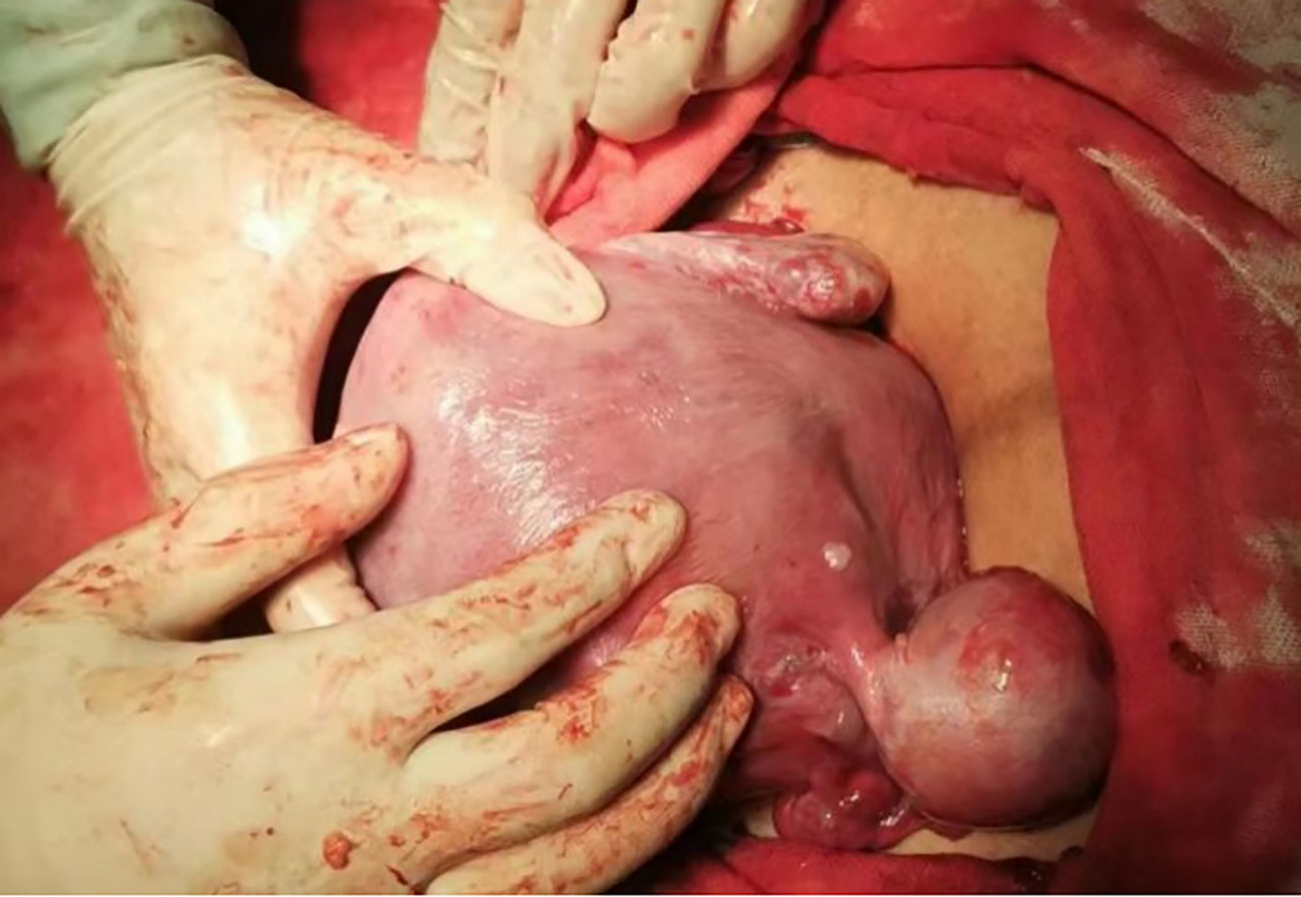
Intraoperative gross appearance of a mature cystic teratoma with a carcinoid tumor (trabecular type).

The initial procedure consisted of cesarean section with left ovarian cystectomy (**Figure 2**). Before the second surgery, a systemic evaluation was performed to exclude metastasis. Contrast-enhanced pelvic magnetic imaging (MRI), chest computed tomography (CT), abdominal ultrasound, and serum tumor markers (CA125 and AFP) were all unremarkable. Because the patient was in the postpartum breastfeeding period and had no symptoms suggestive of metastasis, positron emission tomography-computed tomography (PET-CT) was not performed at that time. A second surgery–single-port laparoscopic left adnexectomy with concurrent myomectomy–was performed six weeks later following the diagnosis of a carcinoid tumor. Systematic lymphadenectomy was not performed based on NCCN and ESMO guidelines for low-grade early-stage ovarian neuroendocrine tumors, the intraoperative absence of enlarged lymph nodes, and the patient’s preference to minimize surgical morbidity. Final pathology confirmed the absence of residual tumor.

### Outcome

The neonate weighed 3620 g and had Apgar scores of 10. Maternal recovery was uneventful. Postoperative tumor markers were normal, and pelvic MRI showed no recurrence. The patient has been followed for 18 months after the initial surgery (16 months after completion surgery) with no evidence of recurrence or metastasis. The follow-up protocol included clinical examination, serum tumor marker assessment (including chromogranin A, which was measured postoperatively and found to be within normal limits), and pelvic imaging every six months during the first two years, followed by annual surveillance thereafter. The patient was also educated regarding symptoms suggestive of carcinoid syndrome.

## Discussion

This case illustrates the occult nature and diagnostic challenges of ovarian carcinoid during pregnancy. Ovarian carcinoids are often associated with teratomas and are classified into insular, trabecular, mucinous, and strumal subtypes (4). Neuroendocrine markers aid in establishing the diagnosis. Differential diagnoses include metastatic carcinoid and other ovarian tumors (5). In our case, the presence of an associated teratoma, unilateral involvement, and the absence of metastatic disease on imaging findings (pelvic MRI, chest CT, abdominal ultrasound, and serum tumor markers) effectively excluded metastatic disease, confirming the diagnosis of primary ovarian carcinoid.

Surgical resection is the mainstay of treatment, with an excellent prognosis reported in patients with early-stage disease. Although the lesion was minute, adnexectomy was performed in accordance with oncological principles. The decision was based on NCCN and ESMO guidelines recommendations advocating complete resection for malignant ovarian neoplasms, the need for definitive staging (which confirmed FIGO IA), and the patient’s completed childbearing status. For patients wishing to preserve future fertility, a fertility-sparing approach with comprehensive staging may be appropriate, and lymphadenectomy may be omitted in low-grade tumors (6).

The low Ki-67 index in this case further supported a favorable prognosis. The final FIGO stage was IA (pT1a cN0 M0), based on tumor confinement to the ovary, an intact capsule, negative peritoneal cytology, and the absence of metastatic disease on imaging findings. This early stage, combined with complete resection and low-grade histology, supports the excellent prognosis observed in this patient.

Our case is demonstrates two particularly unique features. First, the carcinoid focus measured only 1.2 mm, making it one of the smallest ovarian carcinoid foci reported within an ovarian teratoma during pregnancy and explaining its completely occult prenatal presentation. Second, the staged surgical approach—cystectomy during cesarean section followed by definitive adnexectomy after multidisciplinary discussion—allowed for pathological confirmation before radical intervention, a strategy rarely documented in the literature.

Role of multidisciplinary team (MDT): The management of this patient involved an MDT consisting of specialists in obstetrics and gynecology, pathology, radiology, and oncology. The MDT played key roles in several aspects of care: (1) confirming the diagnosis of primary ovarian carcinoid (trabecular type) through combined review of pathological slides and imaging data; (2) reaching a consensus that no postoperative adjuvant chemotherapy or radiotherapy was indicated; and (3) developing a structured stratified follow-up protocol. MDT collaboration is essential for the optimal management of rare pregnancy-associated ovarian tumors.

Tumor markers for neuroendocrine tumors: The most specific serum biomarkers include chromogranin A (CgA) and urine 5-hydroxyindoleacetic acid (5-HIAA). CgA is the first-line serum biomarker for both diagnosis and follow-up of neuroendocrine tumors. Almost all carcinoids secrete CgA, and serum CgA levels reflect tumor burden. In our patient, CgA levels remained within the normal range both preoperatively and during follow-up. CA125 and AFP levels were non-specific but were also within normal limits (CA125: 19.38 U/mL; AFP: 4.34 ng/mL). We recommend routine measurement of CgA and 24-h urine 5-HIAA as baseline and follow-up indicators in future similar cases.

Indications for PET-CT in carcinoid tumors: According to ESMO guidelines, PET-CT is primarily indicated in patients with neuroendocrine tumors of unknown primary, suspected metastasis or recurrence, and atypical or high-grade neuroendocrine tumors. Our patient had a low-grade (G1) trabecular subtype with completely negative systemic imaging findings and therefore did not meet indications for PET-CT. Additionally, the patient was in the postpartum breastfeeding period, and the potential radiation exposure risk to breastfeeding was considered; therefore, PET-CT was not performed.

Comparison with previously reported cases: Primary ovarian carcinoid during pregnancy is extremely rare, with only isolated case reports available in the literature. Our case shares similarities with previously reported cases, including association with teratoma, trabecular subtype, low Ki-67 index, and favorable prognosis. However, it also demonstrates unique features. First, the carcinoid focus measured only 1.2 mm, which may represent the smallest reported ovarian carcinoid focus to date, explaining its prenatal occult nature. Second, the staged surgical strategy (cystectomy during cesarean section followed by completion adnexectomy) has rarely been documented, allowing pathological confirmation before radical intervention.

## Conclusion

Ovarian carcinoid during pregnancy is rare and can present as an occult tumor undetectable on prenatal ultrasound. Complete surgical resection of early-stage disease (FIGO IA) offers an excellent prognosis, as demonstrated by the absence of recurrence in our patient after 18 months of follow-up. Clinicians should maintain vigilance for adnexal masses during pregnancy, and intraoperative inspection and postoperative pathology are crucial for diagnosis. Multidisciplinary collaboration and staged surgical planning are recommended for optimal management and outcomes in these rare cases.

## Data Availability

The original contributions presented in the study are included in the article/supplementary material. Further inquiries can be directed to the corresponding author.
